# Supplementary material for: Disparities in use modalities among adults who currently use cannabis, 2022–2023
Source: J Cannabis Res. 2025 May 16;7:26. doi: 10.1186/s42238-025-00283-x (PMC12082860; doi:10.1186/s42238-025-00283-x)
Supplement: Supplementary file 1 — Supplementary Material 1. [file 42238_2025_283_MOESM1_ESM.pdf]

Disparities in use modalities among adults who currently use cannabis, 2022-2023

Supplementary tables S1-S5

Meman Diaby[1], Osayande Agbonlahor[2], Bethany Shorey Fennell[3,4], Joy L. Hart[5,6], Delvon T. Mattingly[1,7]

[1] Center for Health, Engagement, and Transformation, College of Medicine, University of Kentucky, Lexington, KY, USA; [2] Department of Preventive Medicine, University of Mississippi Medical Center, Jackson, Mississippi, USA; [3] Department of Family & Community Medicine, College of Medicine, University of Kentucky, Lexington, KY, USA; [4] Markey Cancer Center, College of Medicine, University of Kentucky, Lexington, KY, USA, [5] Department of Communication, College of Arts and Sciences, University of Louisville, Louisville, KY, USA; [6] Christina Lee Brown Envirome Institute, School of Medicine, Louisville, KY, USA; [7] Department of Behavioral Science, College of Medicine, University of Kentucky, Lexington, KY, USA

Table S1. Multivariable logistic regression estimating the association between sociodemographic, mental health, and substance use characteristics, and cannabis use modalities among adults who currently use cannabis (n=16,999)

	Cannabis use modalities, AOR (95% CI) [a]						
**Participant characteristics**	Smoking cannabis	Vaping cannabis	Dabbing cannabis	Consuming edibles	Taking pills	Absorbing sublingually/orally [b]	Applying topicals
Age in years (ref: 18-25)							
26-34	0.89 (0.76, 1.05)	**0.68 (0.61, 0.77)**	**0.68 (0.57, 0.81)**	0.96 (0.80, 1.15)	1.18 (0.60, 2.29)	1.24 (0.85, 1.82)	**1.51 (1.16, 1.98)**
35-49	0.77 (0.62, 0.94)	**0.52 (0.45, 0.59)**	**0.49 (0.40, 0.60)**	1.08 (0.92, 1.27)	2.2 (1.25, 3.84)	**1.95 (1.37, 2.77)**	**2.66 (1.99, 3.55)**
50+	**0.73 (0.59, 0.90)**	**0.25 (0.20, 0.31)**	**0.17 (0.11, 0.25)**	**0.76 (0.63, 0.92)**	1.87 (1.01, 3.47)	**2.57 (1.65, 4.00)**	**2.67 (1.81, 3.94)**
Sex (ref: male)							
Female	**0.71 (0.61, 0.82)**	**0.81 (0.71, 0.92)**	**0.70 (0.59, 0.83)**	**1.26 (1.11, 1.43)**	1.05 (0.66, 1.68)	1.19 (0.93, 1.52)	**2.84 (2.17, 3.72)**
Race and ethnicity (ref: non-Hispanic White)							
Hispanic	1.07 (0.88, 1.30)	0.83 (0.70, 0.99)	0.77 (0.61, 0.97)	**0.72 (0.61, 0.86)**	0.69 (0.42, 1.16)	0.84 (0.56, 1.28)	1.19 (0.89, 1.61)
Non-Hispanic Black	**2.05 (1.51, 2.80)**	**0.35 (0.28, 0.44)**	**0.38 (0.28, 0.52)**	**0.68 (0.58, 0.80)**	0.60 (0.37, 0.99)	**0.26 (0.14, 0.49)**	0.93 (0.62, 1.41)
Non-Hispanic multiracial	**1.83 (1.38, 2.43)**	0.83 (0.65, 1.08)	1.08 (0.84, 1.38)	0.89 (0.67, 1.20)	1.43 (0.42, 4.91)	0.95 (0.52, 1.75)	1.38 (0.84, 2.28)
Another non-Hispanic race [c]	1.19 (0.77, 1.84)	**0.63 (0.46, 0.85)**	0.89 (0.57, 1.40)	0.66 (0.44, 0.98)	0.71 (0.24, 2.10)	0.84 (0.42, 1.65)	0.76 (0.34, 1.70)
Sexual orientation (ref: heterosexual)							
LGB+ [d]	0.93 (0.77, 1.14)	**1.28 (1.12, 1.46)**	1.15 (0.98, 1.34)	**1.44 (1.25, 1.66)**	**1.81 (1.21, 2.70)**	1.51 (1.03, 2.23)	1.25 (0.93, 1.68)
Educational attainment (ref: less than high school)							
High school graduate	0.95 (0.68, 1.32)	**1.41 (1.16, 1.72)**	1.17 (0.91, 1.49)	**1.40 (1.14, 1.72)**	0.83 (0.40, 1.71)	**2.00 (1.23, 3.25)**	1.40 (0.84, 2.34)
Some college	**0.65 (0.48, 0.88)**	**1.46 (1.20, 1.77)**	0.86 (0.69, 1.07)	**1.95 (1.54, 2.48)**	1.29 (0.66, 2.52)	**2.41 (1.49, 3.90)**	1.81 (1.11, 2.95)
College graduate or more	**0.39 (0.30, 0.52)**	1.37 (1.08, 1.74)	**0.52 (0.40, 0.67)**	**3.31 (2.53, 4.32)**	1.66 (0.85, 3.24)	**3.31 (2.02, 5.43)**	1.16 (0.65, 2.07)
Annual household income (ref: less than $20,000)							
$20,000 to $49,999	0.75 (0.59, 0.95)	1.13 (0.96, 1.33)	1.08 (0.88, 1.33)	1.20 (1.00, 1.43)	1.36 (0.78, 2.37)	1.54 (1.08, 2.21)	1.11 (0.79, 1.56)
$50,000 to $74,999	**0.65 (0.49, 0.86)**	**1.33 (1.12, 1.58)**	0.85 (0.66, 1.10)	1.19 (0.97, 1.46)	1.68 (0.64, 4.40)	1.36 (0.83, 2.22)	1.27 (0.84, 1.91)
$75,000 or more	**0.51 (0.39, 0.65)**	**1.39 (1.15, 1.67)**	0.78 (0.63, 0.97)	**1.70 (1.40, 2.06)**	1.58 (1.10, 2.26)	1.24 (0.82, 1.90)	1.15 (0.77, 1.71)
Metropolitan status (ref: large metropolitan)							
Small metropolitan	0.93 (0.78, 1.10)	0.99 (0.87, 1.13)	1.28 (1.06, 1.55)	1.08 (0.94, 1.24)	1.05 (0.73, 1.53)	0.99 (0.76, 1.29)	1.39 (1.06, 1.81)
Non-metropolitan	1.05 (0.81, 1.35)	1.03 (0.85, 1.26)	1.31 (1.01, 1.72)	1.15 (0.97, 1.37)	0.99 (0.58, 1.70)	1.05 (0.70, 1.57)	1.37 (0.96, 1.96)
State with medical cannabis laws (ref: No)							
Yes	1.02 (0.85, 1.21)	1.07 (0.93, 1.23)	1.15 (0.94, 1.41)	1.08 (0.92, 1.28)	**2.89 (1.56, 5.38)**	1.53 (0.99, 2.38)	**1.50 (1.16, 1.95)**
Current tobacco product use (ref: No)							
Yes	**2.19 (1.86, 2.57)**	0.92 (0.80, 1.06)	1.28 (1.05, 1.56)	**0.81 (0.71, 0.92)**	0.96 (0.58, 1.59)	0.99 (0.75, 1.32)	0.75 (0.59, 0.95)
Current alcohol use (ref: No)							
Yes	0.91 (0.75, 1.10)	0.94 (0.80, 1.10)	**0.78 (0.67, 0.91)**	1.17 (1.04, 1.32)	0.56 (0.37, 0.86)	0.76 (0.54, 1.06)	0.90 (0.71, 1.14)
Current illicit drug use other than cannabis (ref: No)							
Yes	**1.56 (1.19, 2.03)**	**1.82 (1.52, 2.17)**	**1.91 (1.57, 2.33)**	**1.39 (1.18, 1.63)**	1.55 (1.04, 2.33)	1.16 (0.79, 1.71)	1.26 (0.89, 1.79)
Current psychological distress (ref: No)							
Yes	1.14 (0.94, 1.38)	**1.36 (1.17, 1.59)**	**1.41 (1.19, 1.68)**	1.25 (1.06, 1.47)	1.40 (0.84, 2.33)	1.54 (1.12, 2.12)	**1.43 (1.19, 1.71)**

Bolded adjusted odds ratios and 95% confidence intervals indicate statistical significance (p<0.00625)
AORs and 95% CIs not bolded despite appearing statistically significant did not remain significant after Bonferroni correction
[a] Odds ratios are adjusted for age, sex, race and ethnicity, sexual orientation, educational attainment, annual household income, metropolitan status, state medical cannabis laws status, current tobacco product use, current alcohol use, current alcohol use, current illicit drug use other than cannabis, and current psychological distress
[b] Using drops/strips/lozenges/sprays
[c] Another non-Hispanic race includes respondents who identified as American Indian/Alaska Native, Native Hawaiian/other Pacific Islander, or Asian
[d] LGB+ includes participants who identified as gay, lesbian, or bisexual in both surveys, as well as those who selected "I use a different term," "I am not sure about my sexual identity," or "I do not know what this question is asking" in the 2023 NSDUH survey

Table S2. Multivariable logistic regression estimating the association between sociodemographic, mental health, and substance use characteristics, and blunt use among adults who currently use cannabis (n=12,355)

**Participant characteristics**	AOR (95% CI) [a]
Age in years (ref: 18-25)	
26-34	0.81 (0.66, 1.00)
35-49	**0.72 (0.60, 0.86)**
50+	**0.28 (0.21, 0.38)**
Sex (ref: male)	
Female	1.22 (1.05, 1.43)
Race and ethnicity (ref: non-Hispanic White)	
Hispanic	**1.57 (1.23, 2.01)**
Non-Hispanic Black	**5.68 (4.54, 7.10)**
Non-Hispanic multiracial	1.67 (1.15, 2.43)
Another non-Hispanic race [b]	1.59 (1.11, 2.27)
Sexual orientation (ref: heterosexual)	
LGB+ [c]	0.83 (0.70, 0.98)
Educational attainment (ref: less than high school)	
High school graduate	1.06 (0.84, 1.34)
Some college	**0.71 (0.56, 0.89)**
College graduate or more	**0.30 (0.23, 0.40)**
Annual household income (ref: less than $20,000)	
$20,000 to $49,999	0.91 (0.77, 1.08)
$50,000 to $74,999	0.83 (0.64, 1.07)
$75,000 or more	**0.68 (0.55, 0.85)**
Metropolitan status (ref: large metropolitan)	
Small metropolitan	1.02 (0.86, 1.20)
Non-metropolitan	1.20 (0.93, 1.54)
State with medical cannabis laws (ref: No)	
Yes	0.89 (0.75, 1.04)
Current tobacco product use (ref: No)	
Yes	**2.15 (1.89, 2.44)**
Current alcohol use (ref: No)	
Yes	1.18 (1.01, 1.39)
Current illicit drug use other than cannabis (ref: No)	
Yes	1.32 (1.07, 1.64)
Current psychological distress (ref: No)	
Yes	1.39 (1.10, 1.76)

Bolded adjusted odds ratios and 95% confidence intervals indicate statistical significance ($p<0.00625$)
AORs and 95% CIs not bolded despite appearing statistically significant did not remain significant after Bonferroni correction
[a] Odds ratios are adjusted for age, sex, race and ethnicity, sexual orientation, educational attainment, annual household income, metropolitan status, state medical cannabis laws status, current tobacco product use, current alcohol use, current alcohol use, current illicit drug use other than cannabis, and current psychological distress
[b] Another non-Hispanic race includes respondents who identified as American Indian/Alaska Native, Native Hawaiian/other Pacific Islander, or Asian
[c] LGB+ includes participants who identified as gay, lesbian, or bisexual in both surveys, as well as those who selected "I use a different term," "I am not sure about my sexual identity," or "I do not know what this question is asking" in the 2023 NSDUH survey

Table S3. Factors associated with the number of cannabis use modalities analysis among adults who currently use cannabis: modified Poisson regression (n=16,992)

**Participant characteristics**	Prevalence ratios (95%CI) [a]
Age in years (ref: 18-25)	
26-34	**0.93 (0.90, 0.96)**
35-49	**0.91 (0.87, 0.94)**
50+	**0.74 (0.70, 0.77)**
Sex (ref: male)	
Female	0.98 (0.95, 1.01)
Race and ethnicity (ref: non-Hispanic White)	
Hispanic	**0.92 (0.89, 0.96)**
Non-Hispanic Black	**0.81 (0.77, 0.85)**
Non-Hispanic multiracial	1.03 (0.96, 1.11)
Another non-Hispanic race [b]	0.90 (0.83, 0.98)
Sexual orientation (ref: heterosexual)	
LGB+ [c]	**1.13 (1.09, 1.17)**
Educational attainment (ref: less than high school)	
High school graduate	**1.08 (1.03, 1.14)**
Some college	**1.09 (1.05, 1.14)**
College graduate or more	1.05 (1.00, 1.11)
Annual household income (ref: less than $20,000)	
$20,000 to $49,999	1.03 (0.99, 1.07)
$50,000 to $74,999	1.01 (0.96, 1.06)
$75,000 or more	1.02 (0.98, 1.05)
Metropolitan status (ref: large metropolitan)	
Small metropolitan	1.03 (1.00, 1.06)
Non-metropolitan	1.06 (1.00, 1.11)
State with medical cannabis laws (ref: No)	
Yes	**1.05 (1.02, 1.09)**

Bolded adjusted odds ratios and 95% confidence intervals indicate statistical significance (p<0.05)

[a] Prevalence ratios are adjusted for age, sex, race and ethnicity, sexual orientation, educational attainment, annual household income, metropolitan status, and state medical cannabis laws status

[b] Another non-Hispanic race includes respondents who identified as American Indian/Alaska Native, Native Hawaiian/other Pacific Islander, or Asian

[c] LGB+ includes participants who identified as gay, lesbian, or bisexual in both surveys, as well as those who selected "I use a different term," "I am not sure about my sexual identity," or "I do not know what this question is asking" in the 2023 NSDUH survey

Table S4. Multivariable logistic regression estimating the association between sociodemographic characteristics, state medical cannabis laws status, and cannabis use modalities among adults aged 18-25 who currently use cannabis (n= 6,768)

	Cannabis use modalities, AOR (95% CI) [a]						
**Participant characteristics**	Smoking cannabis	Vaping cannabis	Dabbing cannabis	Consuming edibles	Taking pills	Absorbing sublingually/orally [b]	Applying topicals
Sex (ref: male)							
Female	0.97 (0.78, 1.22)	0.79 (0.66, 0.95)	**0.75 (0.63, 0.89)**	1.20 (1.01, 1.43)	0.79 (0.34, 1.83)	0.79 (0.47, 1.34)	**3.23 (1.67, 6.25)**
Race and ethnicity (ref: non-Hispanic White)							
Hispanic	1.07 (0.76, 1.51)	0.84 (0.65, 1.09)	0.82 (0.63, 1.05)	0.77 (0.60, 0.98)	0.92 (0.37, 2.29)	1.66 (0.81, 3.38)	1.41 (0.81, 2.46)
Non-Hispanic Black	**2.40 (1.56, 3.69)**	**0.34 (0.26, 0.45)**	**0.43 (0.30, 0.63)**	**0.62 (0.48, 0.81)**	1.02 (0.45, 0.29)	0.46 (0.15, 1.36)	1.15 (0.62, 2.11)
Non-Hispanic multiracial	2.25 (1.20, 4.23)	0.88 (0.62, 1.25)	1.17 (0.80, 1.71)	**0.62 (0.46, 0.83)**	0.22 (0.03, 1.85)	1.26 (0.38, 4.18)	1.90 (1.01, 3.58)
Another non-Hispanic race [c]	0.75 (0.39, 1.43)	0.83 (0.56, 1.24)	**0.46 (0.30, 0.71)**	0.58 (0.35, 0.94)	**0.15 (0.05, 0.47)**	0.66 (0.11, 3.87)	0.97 (0.40, 2.35)
Sexual orientation (ref: heterosexual)							
LGB+ [d]	0.76 (0.60, 0.95)	**1.23 (1.07, 1.42)**	1.17 (0.95, 1.46)	**1.46 (1.20, 1.77)**	3.79 (1.46, 9.85)	2.19 (1.07, 4.47)	1.59 (0.91, 2.78)
Educational attainment (ref: less than high school)							
High school graduate	0.94 (0.61, 1.45)	1.21 (0.91, 1.61)	0.87 (0.68, 1.13)	1.17 (0.91, 1.51)	0.82 (0.24, 2.77)	0.97 (0.46, 2.01)	0.96 (0.53, 1.75)
Some college	0.66 (0.43, 1.00)	1.33 (1.00, 1.77)	**0.64 (0.49, 0.83)**	**1.86 (1.41, 2.44)**	1.17 (0.43, 3.19)	0.88 (0.41, 1.92)	0.63 (0.34, 1.16)
College graduate or more	**0.39 (0.24, 0.64)**	0.98 (0.73, 1.34)	**0.43 (0.32, 0.59)**	**2.44 (1.78, 3.35)**	0.66 (0.23, 1.85)	1.16 (0.47, 2.86)	0.41 (0.18, 0.95)
Annual household income (ref: less than $20,000)							
$20,000 to $49,999	0.72 (0.50, 1.04)	1.06 (0.89, 1.27)	1.14 (0.90, 1.44)	0.96 (0.77, 1.20)	0.94 (0.36, 2.48)	1.04 (0.48, 2.25)	0.88 (0.48, 1.62)
$50,000 to $74,999	0.78 (0.55, 1.10)	1.33 (1.05, 1.70)	0.76 (0.59, 0.99)	0.90 (0.67, 1.21)	0.95 (0.24, 3.81)	0.92 (0.37, 2.30)	0.93 (0.51, 1.73)
$75,000 or more	0.76 (0.56, 1.05)	**1.48 (1.23, 1.79)**	1.05 (0.80, 1.37)	1.04 (0.81, 1.32)	0.97 (0.35, 2.64)	1.20 (0.56, 2.58)	0.79 (0.44, 1.41)
Metropolitan status (ref: large metropolitan)							
Small metropolitan	0.76 (0.57, 1.02)	1.09 (0.91, 1.31)	1.33 (1.08, 1.64)	1.04 (0.84, 1.28)	1.03 (0.38, 2.76)	1.24 (0.67, 2.29)	0.90 (0.62, 1.33)
Non-metropolitan	0.92 (0.69, 1.23)	0.90 (0.69, 1.18)	1.25 (0.92, 1.70)	0.97 (0.76, 1.25)	1.06 (0.40, 2.79)	1.12 (0.49, 2.54)	1.09 (0.59, 2.02)
State with medical cannabis laws (ref: No)							
Yes	0.84 (0.65, 1.09)	0.95 (0.76, 1.18)	1.20 (0.95, 1.52)	1.29 (1.03, 1.61)	2.79 (1.14, 6.82)	2.23 (1.22, 4.08)	1.78 (1.00, 3.19)

Bolded adjusted odds ratios and 95% confidence intervals indicate statistical significance ($p<0.00625$)
AORs and 95% CIs not bolded despite appearing statistically significant did not remain significant after Bonferroni correction
[a] Odds ratios are adjusted for sex, race and ethnicity, sexual orientation, educational attainment, annual household income, metropolitan status, and state medical cannabis laws status
[b] Using drops/strips/lozenges/sprays
[c] Another non-Hispanic race includes respondents who identified as American Indian/Alaska Native, Native Hawaiian/other Pacific Islander, or Asian
[d] LGB+ includes participants who identified as gay, lesbian, or bisexual in both surveys, as well as those who selected "I use a different term," "I am not sure about my sexual identity," or "I do not know what this question is asking" in the 2023 NSDUH survey

Table S5. Multivariable logistic regression estimating the association between sociodemographic characteristics, state medical cannabis laws status, and blunt use among adults aged 18-25 who currently use cannabis (n=5,300)

**Participant characteristics**	AOR (95% CI) [a]
Sex (ref: male)	
Female	1.05 (0.86, 1.28)
Race and ethnicity (ref: non-Hispanic White)	
Hispanic	1.03 (0.74, 1.44)
Non-Hispanic Black	**3.77 (2.74, 5.19)**
Non-Hispanic multiracial	1.22 (0.79, 1.89)
Another non-Hispanic race [b]	0.87 (0.53, 1.45)
Sexual orientation (ref: heterosexual)	
LGB+ [c]	0.88 (0.71, 1.09)
Educational attainment (ref: less than high school)	
High school graduate	0.99 (0.72, 1.35)
Some college	**0.62 (0.47, 0.81)**
College graduate or more	**0.28 (0.19, 0.41)**
Annual household income (ref: less than $20,000)	
$20,000 to $49,999	0.90 (0.70, 1.14)
$50,000 to $74,999	0.69 (0.49, 0.98)
$75,000 or more	0.83 (0.64, 1.07)
Metropolitan status (ref: large metropolitan)	
Small metropolitan	1.15 (0.92, 1.43)
Non-metropolitan	1.20 (0.87, 1.66)
State with medical cannabis laws (ref: No)	
Yes	0.79 (0.64, 0.99)

Bolded adjusted odds ratios and 95% confidence intervals indicate statistical significance (p<0.00625)
AORs and 95% CIs not bolded despite appearing statistically significant did not remain significant after Bonferroni correction
[a] Odds ratios are adjusted for sex, race and ethnicity, sexual orientation, educational attainment, annual household income, metropolitan status, and state medical cannabis laws status
[b] Another non-Hispanic race includes respondents who identified as American Indian/Alaska Native, Native Hawaiian/other Pacific Islander, or Asian
[c] LGB+ includes participants who identified as gay, lesbian, or bisexual in both surveys, as well as those who selected "I use a different term," "I am not sure about my sexual identity," or "I do not know what this question is asking" in the 2023 NSDUH survey
